# The prediction of acute ischemic stroke patients’ long-term functional outcomes treated with bridging therapy

**DOI:** 10.1186/s12883-020-1610-1

**Published:** 2020-01-16

**Authors:** Yu-Jun Chang, Chi-Kuang Liu, Wen-Pei Wu, Shih-Chun Wang, Wei-Liang Chen, Chih-Ming Lin

**Affiliations:** 1 Epidemiology and Biostatistics Center, Changhua Christian Hospital, Changhua, Taiwan; 2 Department of Medical Imaging, Changhua Christian Hospital, Changhua, Taiwan; 3 Department of Biomedical Imaging and Radiological Sciences, National Yang-Ming University, Taipei, Taiwan; 4 Department of Neurology, Changhua Christian Hospital, Changhua, Taiwan; 5 Department of Social Work and Child Welfare, Providence University, Taichung, Taiwan; 6 Department of Medicinal Botanicals and Health Applications, Da-Yeh University, Changhua County, Taiwan

**Keywords:** Carotid duplex, Acute ischemic stroke, Thrombolysis therapy, Intra-arterial thrombectomy, Modified Rankin scale, Barthel index

## Abstract

**Background:**

Intravenous thrombolysis therapy (IVT) bridged with intra-arterial thrombectomy (IAT) has recently been recommended as favorable treatment option to ensure that the thrombolytic effect is delivered to the affected region for acute ischemic stroke patients. However, there remains a lack of studies reporting outcome prediction in this group of patients. In this study, we aimed to identify indicators from baseline data that could be used for early prediction of long-term functional outcomes.

**Methods:**

This retrospective single center cohort study included acute ischemic stroke (AIS) patients (*n* = 92) who received IVT and IAT. Functional outcomes were assessed by the National Institute of Health Stroke Scale (NIHSS), modified Rankin Scale (mRS) and Barthel Index. We investigated the relationship between functional outcomes at one-year post-procedure and potential predictors such as occlusion site, modified thrombolysis in cerebral infarction (mTICI) score following the IVT/IAT procedure, and degree of stenosis measured by carotid duplex.

**Results:**

67.4% of the studied patients had satisfactory outcomes with mTICI grades of 2b or 3. From baseline to one-year post-procedure, the NIHSS score improved in 88.0%, the mRS score improved in 69.6%, and the Barthel index improved with 59.8%. Patients with internal carotid artery (ICA) or vertebral artery (VA) stenosis detected by carotid duplex had significantly poorer functional outcomes, measured by the mRS score and Barthel index. In patients with a satisfactory mTICI grade, improvement in the mRS score was only observed in 60.0% of patients with ICA stenosis, compared to 93.8% without ICA stenosis. The VA stenosis was the most significant factor associated with the improvement of mRS (OR = 0.08; 95% CI: 0.01–0.63; *P* = 0.017) and Barthel Index (OR = 0.06; 95% CI: 0.01–0.47; *P* = 0.008) in multiple regression analysis.

**Conclusions:**

ICA or VA stenosis detected by carotid duplex could serve as predictors of significantly poorer functional outcomes in stroke patients treated with bridging therapy; they might be useful clinical markers, particularly as stenosis could be detected by a non-invasive and portable method.

## Background

The current American Heart and Stroke Associations’ (AHA/ASA) guidelines recommend with level of evidence A that “patients should receive mechanical thrombectomy with a stent retriever if they meet all the following criteria: (1) pre-stroke modified Rankin Scale (mRS) score of 0 to 1; (2) causative occlusion of the internal carotid artery (ICA) or middle cerebral artery (MCA) segment 1 (M1); (3) age ≥ 18 years; (4) National Institute of Health Stroke Scale (NIHSS) score ≥ 6; (5) Alberta Stroke Program Early CT Score (ASPECTS) ≥ 6; and (6) treatment can be initiated (groin puncture) within 6 hours of symptom onset.” This recommendation was based on results from 6 randomized controlled trials of mechanical thrombectomy [1–6]. The guidelines also provide clear guidance that “patients eligible for intravenous alteplase should receive intravenous alteplase even if endovascular therapies are being considered [7].”

With the relatively new treatment option of intravenous thrombolysis therapy (IVT) bridged with intra-arterial thrombectomy (IAT) [8, 9], management of acute ischemic stroke (AIS) has entered a new era that has the potential to further improve patient outcomes. Despite evidence-based recommendations based on the 2018 AHA/ASA guidelines, long-term patient outcomes are yet to be thoroughly examined [10, 11].

While large numbers of studies have reported on effective endovascular treatment, there remains limited evidence for the benefit of the combination of IVT and IAT treatments. A systematic review of 8 studies reported that the mortality did not increase when treatment with IVT was carried out, and mechanical IAT could reduce long-term disability if conducted in the recommended subset of patients with AIS resulting from large vessel occlusion and [7]. However, more evidence is still needed to ascertain whether IVT prior to IAT is superior to IAT alone, or whether there are any surrogate baseline markers that may predict patients’ functional outcomes at earlier time points.

In this retrospective study, we attempted to identify indicators from baseline data of AIS patients that could be used in the early prediction of long-term functional outcomes after IVT and IAT. In addition, we also hypothesized extracranial internal carotid artery (ICA)/vertebral artery (VA) stenosis could influence the outcomes of this group of stroke patients. Therefore, the primary aim of this study was to investigate the relationship between functional outcomes (measured by NIHSS, Barthel Index and mRS) at one-year post-procedure and potential predictors such as intracranial occlusion site and modified thrombolysis in cerebral infarction (mTICI) score following IVT and IAT procedures. Secondly, we also collected the data of the site of stenosis from extracranial carotid arteries detected by the carotid duplex, as well as resistance index derived from the carotid doppler, to determine which parameters are significantly associated with the stroke patients’ long term functionality. Through this study we hope to provide front-line clinicians with practical information in terms of directing favorable outcomes after bridging therapy.

## Methods

### Patient involvement

**Fig. 1:**
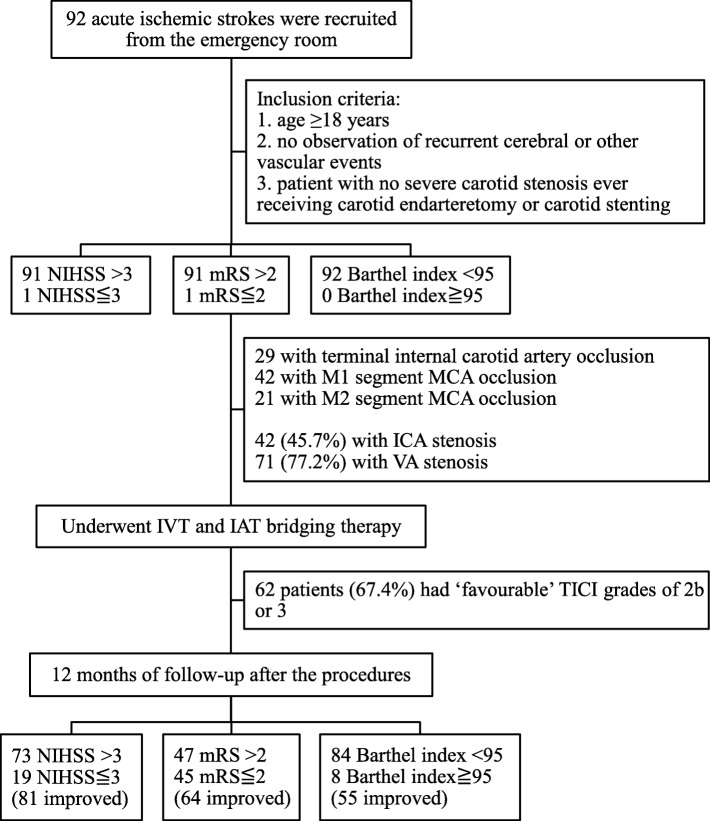
Study flowchart

In this retrospective study, data were collected from the medical records of 92 consecutive acute ischemic stroke (AIS) patients who underwent IVT bridged with IAT therapy during the period January 2015 to April 2017 at the angiography laboratory of the Department of Neuroimaging, Changhua Christian Hospital, Changhua, Taiwan. Originally, 100 patients were recruited in the emergency room. Of these, 8 cases were confirmed to have ophthalmic artery stenosis presenting with the monocular blindness instead of acute ischemic stroke episodes, and were subsequently excluded from the study. All patients included in the study had met pre-defined inclusion and exclusion criteria as followings. The inclusion criteria was: age ≥ 18 years; no observation of recurrent cerebral or other vascular events during the study period; patient with no severe carotid stenosis/vertebral stenosis ever receiving carotid endarterectomy or carotid stenting; completion of at least 12 months of follow-up after thrombectomy procedures. The exclusion criteria was: patients with cerebral primary hemorrhage; those with cerebral arteriovenous malformations or aneurysms; recurrent stroke during the study period; patients demonstrating systemic vascular events or recurrent cerebral vascular episodes (Fig. 1).

Baseline biochemistry and neuroradiological exams were carried out in the emergency room. The clinical information collected was cross-checked to ensure consistency between the original (paper) medical records and the electronic information of the Changhua Christian Hospital 2000 computer-based medical record systems network. The majority of data was extracted by two data extractors (Dr. Chih Ming Lin and Dr. Chi Kuang Liu). The study procedures were approved by the Institutional Review Board of the Changhua Christian Hospital in Changhua, Taiwan (CCH IRB number: 180409).

### Imaging and clinical assessment

#### Cervical carotid ultrasound examination

Cervical carotid artery ultrasound examination (Philips iE33 7-Mhz linear transducer) was performed following patient admission. Cross-sectional B-mode scanning was performed to check for intraluminal plaque material and the longitudinal screening method was adopted to confirm the presence of plaque. The classification of plaques into subtypes 1, 2, 3, or 4 according to the International Classification System [12] was assessed with consensus by two physicians. In case of disagreement between the physicians, a third physician assessed the classification. The intima media thickness of the mid-portion of the common carotid artery (CCA) was measured on the ipsilateral side of the index stroke event. Peak-systolic velocity (PSV), end-diastolic velocity (EDV), and the resistance index (RI; calculated as (PSV-EDV)/PSV) and pulsatility index (PI; (PSV-EDV)/mean of the velocity [1/3PSV+ 2/3EDV]) of the CCA, internal carotid artery (ICA), external carotid artery (ECA), vertebral artery (VA) and ophthalmic artery (OA) were measured bilaterally. The reversal of blood flow in the OA was also measured. Forward flow was defined as blood flow away from the stenotic ipsilateral carotid artery, whereas reverse flow was defined as blood flow into the carotid artery. Plaque index [13] was also calculated automatically by the technicians and machine for both ipsilateral and contralateral to the cerebral lesion site.

#### CT angiography (CTA) / CT perfusion (CTP) protocol

The computed tomographic (CT) stroke protocol was performed on a dual source CT scanner (Siemens Definition Flash). Pre- and post-contrast CT scans of the head with the following parameters were performed: 120 kV (peak) (kVp), 330 mA (auto), 64 × 0.6 mm collimation, 0.28 s/rotation, and table speed of 1 mm/rotation. CT angiography (CTA) was performed from the aortic arch to the vertex with the following parameters: total 60 cc iodinated contrast agent was injected at 5 mL/s (Iohexol, Omnipaque, 350 mg iodine/mL; GE Healthcare, Piscataway, NJ), 5- to 10-s delay, 100/140 kVp, auto mA, 0.28 s/rotation, 0.6-mm-thick sections, and table speed of 4 cm/rotation. CTA data were automatically processed by the technicians, including multiplanar 5 mm maximum intensity projection (MIP) reconstructions and 5-mm axial reformats or CTA source images. The CT perfusion (CTP) technique included 45-s scanning reconstructed at 0.5-s intervals to produce a series of 90 sequential images for each of the 8 sections, covering a total of 40 mm from the basal ganglia to the lateral ventricles. CTP scanning parameters were the following: 80 kVp, 150 mA, total 50 cc iodinated contrast agent injected at 5 mL/s.

CTA/P was conducted at the time of intravenous IVT administration. This is a mandatory standard procedure so the neurointerventionist can evaluate the adequacy of IAT. Perfusion datasets were post-processed on a Siemens Multimodality Workplace Workstation (Siemens Medical, Germany) and mean transit time (MTT), cerebral blood volume (CBV), cerebral blood flow (CBF), and time to peak (TTP) maps, core, penumbra, Tmax, and mismatch regions were also recorded.

### Intervention

#### Intravenous thrombolytic therapy (IVT)

All patients received pre-interventional IVT with recombinant tissue plasminogen activator (rtPA) bridged with IAT treatment. The administration of IVT with rtPA for this study was in accordance with Changhua Christian Hospital protocols at ≤4.5 h after onset of symptoms. Before administration, thorough patient evaluation was carried out by a stroke neurologist and included cerebral CT. The patient’s stroke severity was scored using the National Institute of Health Stroke Scale (NIHSS) score and the inclusion and exclusion criteria [1]. Informed consent was obtained from either the family or the patient in the emergency room setting. Shortly after rtPA IVT therapy, suitability for IAT was evaluated with CTA/P. A neuroradiologist double-checked the patency of the large vessel. The window of time allowed for IAT to the anterior circulatory system was set at ≤8 h after onset of symptoms at our institution [1]. Before the IAT was commenced, patient baseline characteristics were recorded which included demographic information, NIHSS score, mRS score, Barthel index, blood biochemistry, and CTA/P.

#### Angiography and intra-arterial thrombectomy (IAT)

All procedures were conducted by a neurointerventional team in a specialized angiography clinic at the Changhua Christian Hospital, Taiwan. The indications for IAT were findings of major artery stenosis (visualized and confirmed by a neuroradiologist) with location of suitable accessibility for the procedure to be carried out i.e. terminal intracranial internal carotid artery (TICA), first branch of middle cerebral artery (M1) or second branch of middle cerebral artery (M2) [1]. The intra-arterial endovascular thromboaspiration procedure was performed with informed consent from the patient or family.

Under general endotracheal anesthesia, one 9F right femoral sheath was inserted through the right femoral artery and then a Neuron Max 088 catheter (Penumbra Inc., Alameda, CA, USA) with a coaxial JB2 catheter (Cook Medical Inc., Bloomington, IN, USA) was advanced to the common carotid artery or internal carotid artery. A diagnostic cerebral angiogram was performed to confirm the location and extension of the blood clot.

Next, one 8F right femoral sheath was inserted and one 6F NeuroMax long sheath was advanced up to the stenotic site, then one 3Max Penumbra aspiration reperfusion catheter was inserted coaxially via the guiding catheter and navigated to the proximal part of the occluded MCA and then mechanical thrombectomy was performed using the Penumbra aspiration catheter (Penumbra Inc.) or Solitaire™ Platinum revascularization device (Medtronics Inc., Minneapolis, MN, USA). Follow-up Dyna-CT was conducted to determine that there was no apparent hemorrhage or contrast stasis.

The mTICI Score, an indication of immediate outcome, was instantly evaluated by a neuroradiologist [14, 15]. The mTICI Score was defined as follows: 0 = no perfusion; 1 = penetration, but no distal branch filling; 2a = perfusion with incomplete (< 50%) distal branch filling; 2b = perfusion with incomplete (> 50%) distal branch filling; and 3 = full perfusion with filling of all distal branches. Scores of 2b and 3 were defined as a satisfactory result while 0, 1, and 2a were regarded as poor revascularization.

### Outcome measures

Outcome measurement parameters included the NIHSS score, mRS score, and Barthel index [1–6] were re-evaluated at 1 year after the IVT and IAT therapy, conducted in an outpatient clinic setting. The NIHSS is specifically used for measuring the degree of neurological defects (with <=3 defined as mild while > = 25 is defined as severe ischemic stroke episode). The mRS is used for assessing general daily life functionality and independence (with <= 2 defined as independence). Likewise, the Barthel index is used for assessing general daily independence with more detailed items (with > = 60 points indicating favorable life mobility; > = 95 denoting normal or near normal life mobility).

### Statistical analysis

In this study, the mTICI scores were dichotomized and defined as no substantial reperfusion (mTICI 0,1, 2a) vs. substantial reperfusion (mTICI 2b–3). The Student’s t-test was used to compare baseline characteristics and clinical outcomes between both groups. Functional outcomes were assessed by the NIHSS, mRS and Barthel Index. We divided each of the three functional outcomes into two categories with and without improvement after 1 year of tracking and comparison. Univariate analysis was then conducted for the three outcome variables using chi-square or student’s t-test on each variable. The proximal ICA/VA stenosis and reperfusion had interaction effects on functional outcomes in patients. Therefore, we further performed stratified analyses to evaluate the relationship between arterial stenosis and functional outcomes with or without reperfusion. Finally, we use logistic regressions to predict factors associated with three different measurements of functional outcome. The independent variables with a *P*-value of less than 0.05 in the univariate analysis were selected for multivariate analysis. The final model only retained the significant predictors (*P* < 0.05). All the data in this study were analyzed using the IBM SPSS Statistics for Windows, Version 22.0 (IBM Corp., Armonk, NY). *P*-values < 0.05 were considered statistically significant.

## Results

In total, 92 patients met the inclusion criteria and were included in the analysis. Patients had terminal internal carotid artery occlusion (TICA; *n* = 29), M1 segment MCA occlusion (M1; *n* = 42) or M2 segment MCA occlusion (M2; *n* = 21). Seventeen (18.5%) patients had had a previous stroke. Characteristics of patients, tabulated by site of occlusion, are presented in Additional file 1: Table S1 and Table S2. Significantly more male patients were included, and males were more likely to have M2 occlusions than females (81% male versus 19% female). Overall, NIHSS score improved from baseline to one-year post-procedure in 81 (88.0%) patients, mRS score improved in 64 (69.6%) and Barthel Index improved in 55 (59.8%)(Additional file 1: Table S3 and Table 1).

**Table 1:** Demographics and associated parameters for intra-arterial thrombectomy measured by mTICI

					Endovascular mTICI grading									*P*-value
	Total (*n* = 92)				0,1,2a (*n* = 30)					2b or 3 (*n* = 62)				
	Median	IQR			Median	IQR				Median	IQR			
Age (year)	68.0	55.5	–	77.0	64.5	54.0	–	71.0		69.0	56.0	–	79.0	0.255
BMI (kg/m^2^)	25.0	23.2	–	27.5	25.2	22.8	–	26.6		24.8	23.5	–	28.7	0.591
Triglyceride (mg/dL)	95.5	60.0	–	147.0	96.0	67.0	–	143.0		91.5	57.0	–	149.0	0.868
Total cholesterol (mg/dL)	161.5	136.0	–	184.0	157.0	137.0	–	174.0		164.5	136.0	–	185.0	0.608
hSCRP (mg/dL)	2.8	1.2	–	5.8	2.9	1.2	–	9.3		2.7	1.2	–	5.5	0.532
CT ASPECTS-admission	9.0	8.0	–	9.0	8.0	7.0	–	9.0		9.0	8.0	–	9.0	0.320
Ischemic core	16.0	3.0	–	34.0	18.5	0.0	–	39.0		14.0	4.0	–	34.0	0.890
Mismatch	8.7	4.2	–	23.1	5.7	3.6	–	19.6		9.0	4.6	–	23.3	0.121
Perfusion Tmax	119.5	72.0	–	161.0	141.0	71.0	–	174.0		116.0	74.0	–	155.0	0.375
NIHSS-admission	21.0	16.5	–	25.0	21.5	17.0	–	26.0		21.0	16.0	–	25.0	0.716
NIHSS-1 year later	10.0	5.0	–	17.0	10.0	6.0	–	17.0		10.0	5.0	–	17.0	0.783
NIHSS-improvement	8.5	4.0	–	17.0	9.0	2.0	–	19.0		8.0	4.0	–	17.0	0.671
mRS-admission	5.0	4.0	–	5.0	5.0	4.0	–	5.0		5.0	4.0	–	5.0	0.773
mRS-1 year later	3.0	2.0	–	5.0	3.5	2.0	–	6.0		2.5	1.0	–	4.0	0.093
mRS-improvement	2.0	0.0	–	3.0	1.0	−1.0	–	3.0		2.0	1.0	–	3.0	0.136
Barthel index-admission	10.0	5.0	–	20.0	10.0	0.0	–	20.0		10.0	10.0	–	20.0	0.296
Barthel index-1 year later	20.0	5.0	–	45.0	10.0	0.0	–	30.0		20.0	5.0	–	50.0	0.077
Barthel index-improvement	10.0	−2.5	–	27.5	5.0	−10.0	–	20.0		12.5	0.0	–	30.0	0.150
IMT-ipsilateral (mm)	0.8	0.6	–	0.9	0.7	0.6	–	0.8		0.8	0.6	–	0.9	0.211
Plaque index-ipsilateral	1.0	0.0	–	3.5	1.0	0.0	–	4.0		2.0	0.0	–	3.0	0.939
Plaque index-contralateral	2.0	0.0	–	4.0	2.5	0.0	–	4.0		2.0	1.0	–	4.0	0.896

*mTICI* The modified thrombolysis in cerebral infarction, *IQR* Interquartile range is the difference between the first quartile and third quartile. P-value by Mann-Whitney U test; Mismatch is the value form CTA/P, the formula is defined as ischemic region/death core of cerebral parenchyma; *BMI* Body mass index, *hsCRP* high sensitivity C reactive protein, *ASPECTS* The Alberta Stroke Program Early CT Score, *NIHSS* National Institute of Health Stroke Scale, *mRS* modified Rankin Scale, *IMT* Intima media thickness

The mTICI scores, which give an indication of procedure outcome, are presented in Table 1. In total 62 patients (67.4%) had ‘successful’ mTICI grades of 2b or 3. Within these IAT success cases, NIHSS score improved from baseline to one-year post-procedure in 56 (90.3%) patients, mRS score improved in 48 (77.4%) and Barthel index improved in 39 (62.9%). The mean NIHSS, mRS and Barthel Index all improved for both poor and satisfactory mTICI grades. The improvement in mean Barthel Index was significantly higher for patients with a satisfactory mTICI (*P* = 0.024; Table 1).

The majority of patients had a poor NIHSS score on admission with 91 patients (98.9%) scoring > 3, but one-year post-procedure the number of patients with a poor NIHSS score > 3 had significantly reduced to only 73 patients (79.3%) (*P* < 0.001). Similarly, on admission 91 patients had a poor mRS score (> 2), but one-year post-procedure mRS was poor in only 47 patients (51.1%) (P < 0.001). However, only 8 patients (8.7%) had a Barthel index that improved to ≥95 at one-year post-procedure (Fig. 1, Additional file 1: Table S3).

Carotid duplex was used to determine the extent of ICA and VA stenosis. A patient was considered to have ICA/VA stenosis if visualization of stenosis of plaque formation detected by B-mode carotid duplex scanning as well as complimentary data, the resistance index (RI) was ≥0.75, indicating focal stenosis /down- stream stenosis [13]. Overall, 42 patients had ICA stenosis and 71 patients had VA stenosis. Functional outcomes are shown in Table 2. Patients with ICA or VA stenosis had significantly poorer functional outcomes, as measured by mRS and Barthel Index. For patients with a satisfactory IAT outcome, mRS score improved in 93.8% of those with no ICA stenosis and 93.3% of those with no VA stenosis. In comparison, only 60.0 and 72.3% showed improved mRS score if they had ICA or VA stenosis respectively (Table 2).

**Table 2:** Subgroup analyses of the relationship between immediate outcomes of the the IVT/IAT procedure, arterial stenosis, and long-term functional outcomes

Immediate outcome	Arterial stenosis		Total	NIHSS improved		*P*-value	mRS improved		*P*-value	Barthel index improved		*P*-value
				N	%		N	%		N	%	
Unfavorable (2a)		Total	30	25	83.3		16	53.3		16	53.3	
	ICA stenosis	No	18	14	77.8	0.622	10	55.6	0.765	10	55.6	0.765
		Yes (RI ≥0.75)	12	11	91.7		6	50.0		6	50.0	
Favourable (2b,3)		Total	62	56	90.3		48	77.4		39	62.9	
	ICA stenosis	No	32	29	90.6	1.000	30	93.8	0.001	27	84.4	< 0.001
		Yes (RI ≥0.75)	30	27	90.0		18	60.0		12	40.0	
Overall		Total	92	81	88.0		64	69.6		55	59.8	
	ICA stenosis	No	50	43	86.0	0.510	40	80.0	0.018	37	74.0	0.002
		Yes (RI ≥0.75)	42	38	90.5		24	57.1		18	42.9	
Unfavorable (2a)		Total	30	25	83.3		16	53.3		16	53.3	
	VA stenosis	No	6	6	100.0	0.553	6	100.0	0.019	5	83.3	0.175
		Yes (RI ≥0.75)	24	19	79.2		10	41.7		11	45.8	
Favourable (2b,3)		Total	62	56	90.3		48	77.4		39	62.9	
	VA stenosis	No	15	15	100.0	0.321	14	93.3	0.155	15	100.0	0.001
		Yes (RI ≥0.75)	47	41	87.2		34	72.3		24	51.1	
Overall		Total	92	81	88.0		64	69.6		55	59.8	
	VA stenosis	No	21	21	100.0	0.063	20	95.2	0.004	20	95.2	< 0.001
		Yes (RI ≥0.75)	71	60	84.5		44	62.0		35	49.3	

P-value by Chi-square test or Fisher’s exact test where appropriate; *IAT* Intra-arterial thrombectomy, *NIHSS* National Institute of Health Stroke Scale, *mRS* modified Rankin Scale, *ICA* Internal carotid artery, *VA* Vertebral artery

Furthermore, regardless of IAT success, patients with no ICA stenosis were significantly more likely to demonstrate improved functional outcomes compared to those with ICA stenosis, as measured by both mRS (80.0% vs 57.1%, *P* = 0.018) and Barthel Index (74.0% vs 42.9%, *P* = 0.002). Similar results were observed for VA stenosis (*P* = 0.004 and *P* < 0.001 respectively; Table 2).

The results of the multiple logistic regression analysis are presented in Table 3. The VA stenosis (OR = 0.08; 95% CI: 0.01–0.63; *P* = 0.017) was significantly associated with improved mRS at one-year post-procedure. A similar statistical result (OR = 0.06; 95% CI: 0.01–0.47; *P* = 0.008) was observed in the Barthel Index improved patients, indicating that the stenosis of extracranial posterior circulation could have an impact on the patients subsequent functional outcome.

**Table 3:** Results of logistic regression analysis of baseline variables for the prediction of favorable functional outcomes at one-year post-procedure

Dependent variable	Predictor		Univariate analysis (crude)					Multiple analysis (adjusted)				
			Odds ratio	95% CI			*P*-value	Odds ratio	95% CI			*P*-value
NIHSS improved	NIHSS-admission		1.109	1.015	–	1.213	0.023	1.123	1.017	–	1.240	0.021
	Mismatch		0.974	0.952	–	0.998	0.030	0.964	0.934	–	0.995	0.025
	VA PI-ipisilateral		0.692	0.481	–	0.995	0.047	0.673	0.466	–	0.974	0.036
mRS improved	Age		0.972	0.941	–	1.004	0.089					
	NIHSS-admission		0.947	0.887	–	1.011	0.100					
	ICA stenosis	No	1.000									
		Yes (RI > =0.75)	0.333	0.132	–	0.840	0.020					
	VA stenosis	No	1.000					1.000				
		Yes (RI > =0.75)	0.081	0.010	–	0.642	0.017	0.078	0.010	–	0.629	0.017
	Fasting blood glucose	< 100	1.000									
		> = 100	3.867	1.195	–	12.511	0.024					
	Endovascular mTICI grading	2a	1.000					1.000				
		2b or 3	3.000	1.181	–	7.620	0.021	3.154	1.169	–	8.511	0.023
Barthel index improved	Age		0.954	0.923	–	0.986	0.006					
	NIHSS-admission		0.914	0.855	–	0.977	0.008	0.933	0.869	–	1.002	0.057
	MCA stenosis-contralateral	No	1.000									
		Yes	0.295	0.119	–	0.731	0.008					
	Plaque index-ipisilateral		0.773	0.642	–	0.932	0.007	0.799	0.645	–	0.990	0.040
	ICA stenosis	No	1.000									
		Yes (RI > =0.75)	0.264	0.109	–	0.635	0.003					
	VA stenosis	No	1.000					1.000				
		Yes (RI > =0.75)	0.049	0.006	–	0.382	0.004	0.057	0.007	–	0.470	0.008
	Triglyceride	< 150	1.000									
		> = 150	4.014	1.232	–	13.070	0.021					

*NIHSS* National Institute of Health Stroke Scale, *mRS* modified Rankin Scale, *CI* Confidence interval, *ICA* Internal carotid artery, *VA* Vertebral artery, *MCA* Middle cerebral artery, *mTICI* The modified thrombolysis in cerebral infarction

## Discussion

In this retrospective single center cohort study of 92 AIS patients, we observed that the majority of patients (*n* = 62; 67.4%) had satisfactory outcomes with mTICI grades [14] of 2b or 3 following IVT and IAT (Table 1). This success rate is comparable to that found in other studies. For example, Coutinho et al 2017 found that 127/151 (84.1%) patients had mTICI grades of 2b or 3 following IVT and IAT [16]. Similarly, Gerschenfeld et al (2017) [17] reported 84/100 (84.0%) and Kaesmacher et al (2018) [8] reported that 75/160 (46.9%) patients had mTICI grades of 2b or 3 after IVT and IAT treatment. There is limited evidence for longer term outcomes of IVT and IAT treatments in AIS patients [16, 18–20]. However, in all four studies the follow-up was only for 3 months. In our study, long-term functional outcomes following bridging therapy were promising with the majority of patients experiencing improved NIHSS score, mRS score and Barthel Index at one-year post-procedure.

In this study, the use of three different outcome measurements enabled us to gauge the patients’ functionality after receiving the bridging therapy, by considering both daily life activity independence and recovery of neurological deficits. Among the three measuring tools, it was discovered the NIHSS score was the most sensitive in detecting patients’ recovery (88.0%), followed by mRS (69.6%) and Barthel Index (59.8%)(Additional file 1: Table S3 and Table 2). This is likely due to the fact that NIHSS aims to factor in all aspects of neurological defects, while Barthel Index and mRS aim to evaluate general independence and ability of AIS patients. For patients with acute cerebral infarction, after removal of the vascular thrombus, the damaged neurological function will initially improve, but still require further long-term treatment and rehabilitation in order to restore the patient’s muscle strength, so that they can complete various daily activities independently again.

Furthermore, we also found that the improvement of mRS and Barthel index had a strong correlation with ICA and VA stenosis. The improvement rate of the three scales of those who succeeded after IVT and IAT is better than that of unsuccessful ones. However, if they had ICA or VA stenosis, regardless of whether there was success after IVT and IAT, the improvement rate of mRS and Barthel index was less than half of those without stenosis (Table 2).

Through multiple regression analyses we also found that VA stenosis was significantly negatively correlated with an improved mRS and Barthel index. After controlling for other confounding factors, as long as there was no VA stenosis, the odds of improvement were more than 12 times in mRS score and 17 times in Barthel index score higher than for those with stenosis (Table 3). Taken together, this shows that both mRS score and Barthel Index were more likely to have improved at one-year follow-up in those patients without ICA or VA stenosis. As such, ICA and VA stenosis could be a useful indicator to predict outcome in AIS patients. Carotid duplex is a feasible and useful technique for detecting ICA and VA stenosis, especially as it is portable and non-invasive. Carotid duplex may thus help identify patients who will benefit the most from the combined IVT/IAT procedures. The hypothesized explanation for this regard might be due to the “insufficient backup system” (patients with the extrcranial ICA/ VA stenoses) jeopardizing the collateral systems upon patients suffering from the stroke episode regardless of the subsequent bridging therapy. With the intact collateral systems, the bridging therapy could enhance/expedite the recanalization process and decrease the size of penumbra, leading to rosy functional recovery.

To the best of our knowledge, this finding has only been observed in one other study, but this was in patients with extracranial and/or intracranial tandem occlusions of the anterior circulation who were treated with both mechanical thrombectomy and carotid artery stenting. In the study by Maus et al (2018), reperfusion was successful in similar numbers of patients with and without contralateral carotid stenosis, yet those patients with contralateral stenosis > 50% had significantly poorer outcomes (mRS > 2) at 90 days. The researchers concluded that contralateral carotid stenosis > 50% was an independent predictor of poor clinical outcome and postulated that poor collateral flow to affected tissue was the probable cause [21].

It is also interesting to note that in a 2018 analysis of data from the Interventional Management of Stroke (IMS)-III trial, it was found that severe carotid stenosis (> 70%) was associated with increased time to reperfusion following endovascular treatment [22]. Baseline NIHSS was also associated with increased time to reperfusion in this study. This delayed reperfusion is consistent with the findings in our study and may relate to systemic inflammation or platelet debris deposition on the larger artery, as reflected by the delayed Tmax and mismatch (Additional file 1: Table S1 and S2), suggesting there is a greater volume of cerebral tissue that requires reperfusion following endovascular therapy.

The strengths of this study include that while being carried out in only one institution, this analysis benefitted from a therefore standardized approach with very limited treatment-methodology associated variability, IAT procedures were all conducted in the same facility by a small team of physicians, and there was consistency in the collection and recording of data. Weaknesses include that it is a relatively small study with 92 cases only and the study period is merely 1 year after the thrombectomy treatment with retrospective in nature. The stroke patients included in this study were first time ever. The outcome for the recurrent stroke group was unknown. The generalization of the study results might be limited. In addition, post–thrombectomy treatment such as physical rehabilitation and antiplatelet therapy might potentially confound the functional outcomes measured by the NIHSS, Barthel Index and mRS. We were unfortunately unable to collect the post-thrombectomy carotid duplex datasets as they were incomplete and insufficient for comparison. The changes in resistance index is not able to be directly compared as there was no comparison (control) group that had IAT or IVT alone, in order to determine whether IVT and IAT in combination confers some advantages in patients with certain characteristics. Therefore, additional prospective large-scale clinical trials with a focus on the extracranial and intracranial resistance index changes will be required in the future to overcome the limitations of the present study.

## Conclusions

Bridging therapy (IVT and IAT) was a major treatment option especially for stroke patients within therapeutic window. However, not all successful procedures (with mTICI of 2b or 3) had good long-term outcomes. Stroke patients with ICA or VA stenosis had significantly poorer functionality following bridging therapy. Using noninvasive examination carotid duplex to assess ICA and VA stenosis prior to treatment may assist in defining the patient group who will benefit the most from this treatment and in predicting outcomes.

## Supplementary information

**Additional file 1:** **Table S1** Characteristics of patients with differing occlusion sites: TICA, M1 and M2. **Table S2** Baseline demographic of patients with differing occlusion sites: TICA, M1 and M2. **Table S3** Outcome measurements at baseline and 1 year post-procedure

## Data Availability

The data that support the findings of this study are available from Changhua Christian Hospital but restrictions apply to the availability of these data, which were used under permit for the current study, and so are not publicly available. Data are however available from the authors upon reasonable request and with permission of Changhua Christian Hospital.

## Abbreviations

AHA/ASA: American Heart and Stroke Associations
AIS: Acute ischemic stroke
ASPECTS: Alberta Stroke Program Early CT Score
BI: Barthel Index
CBV: Cerebral blood volume
CCA: Common carotid artery
CI: Confidence interval
CT: Computed tomographic
CTA: CT angiography
CTP: CT perfusion
ECA: External carotid artery
EDV: End-diastolic velocity
IAT: Intra-arterial thrombectomy
ICA: Internal carotid artery
IVT: Intravenous thrombolysis therapy
M1: First branch of middle cerebral artery
M2: Second branch of middle cerebral artery
MCA: Middle cerebral artery
MIP: Maximum intensity projection
mRS: modified Rankin Scale
mTICI: modified thrombolysis in cerebral infarction
MTT: Mean transit time
NIHSS: National Institute of Health Stroke Scale
OA: Ophthalmic artery
OR: Odds ratio
P: *p*-value
PI: Pulsatility index
PSV: Peak-systolic velocity
RI: Resistance index
rtPA: recombinant tissue plasminogen activator
TICA: Terminal intracranial internal carotid artery
TTP: Time to peak
VA: Vertebral artery
