# Supplementary material for: The prediction of acute ischemic stroke patients’ long-term functional outcomes treated with bridging therapy
Source: BMC Neurol. 2020 Jan 16;20:22. doi: 10.1186/s12883-020-1610-1 (PMC6966893; doi:10.1186/s12883-020-1610-1)
Supplement: Supplementary file 1 — Additional file 1: Table S1 Characteristics of patients with differing occlusion sites: TICA, M1 and M2. Table S2 Baseline demographic of patients with differing occlusion sites: TICA, M1 and M2. Table S3 Outcome measurements at baseline and 1 year post-procedure [file 12883_2020_1610_MOESM1_ESM.docx]

**Supplementary**

**Table S1** Characteristics of patients with differing occlusion sites: TICA, M1 and M2.

|  | Occlusion site | | | | | | | | | | | | | |  |  |  |
| --- | --- | --- | --- | --- | --- | --- | --- | --- | --- | --- | --- | --- | --- | --- | --- | --- | --- |
|  | Terminal ICA (n=29) | | | |  | M1 (n=42) | | | |  | M2 (n=21) | | | |  | P-value^a^ | P-value^b^ |
|  | Median | IQR | | |  | Median | IQR | | |  | Median | IQR | | |  |  |  |
| Age (year) | 69.0 | 61.0 | - | 76.0 |  | 65.5 | 53.0 | - | 77.0 |  | 68.0 | 59.0 | - | 77.0 |  | 0.752 | 0.802 |
| BMI (kg/m^2^) | 26.0 | 24.3 | - | 31.1 |  | 24.3 | 22.7 | - | 26.5 |  | 25.8 | 23.1 | - | 30.9 |  | 0.050 | 0.281 |
| Triglyceride (mg/dL) | 136.0 | 76.0 | - | 177.0 |  | 86.0 | 59.0 | - | 140.0 |  | 85.0 | 54.0 | - | 122.0 |  | 0.076 | 0.025 |
| Total cholesterol (mg/dL) | 177.0 | 145.0 | - | 202.0 |  | 153.0 | 137.0 | - | 177.0 |  | 159.0 | 136.0 | - | 170.0 |  | 0.112 | 0.063 |
| hSCRP (mg/dL) | 4.4 | 2.7 | - | 7.3 |  | 2.4 | 1.0 | - | 5.6 |  | 2.0 | 1.0 | - | 4.9 |  | 0.054 | 0.032 |
| CT ASPECTS-admission | 8.0 | 7.0 | - | 9.0 |  | 9.0 | 8.0 | - | 9.0 |  | 9.0 | 7.0 | - | 9.0 |  | 0.385 | 0.573 |
| Ischemic core | 11.0 | 4.0 | - | 28.0 |  | 16.5 | 3.0 | - | 34.0 |  | 24.0 | 2.0 | - | 45.0 |  | 0.488 | 0.229 |
| Mismatch | 12.5 | 5.7 | - | 24.6 |  | 8.1 | 4.2 | - | 23.2 |  | 6.1 | 3.2 | - | 12.8 |  | 0.059 | 0.015 |
| Perfusion Tmax | 141.0 | 117.0 | - | 179.0 |  | 114.0 | 74.0 | - | 155.0 |  | 78.0 | 32.0 | - | 128.0 |  | 0.041 | 0.008 |
| NIHSS-admission | 24.0 | 19.0 | - | 28.0 |  | 20.5 | 15.0 | - | 25.0 |  | 21.0 | 17.0 | - | 25.0 |  | 0.110 | 0.213 |
| NIHSS-1 year later | 10.0 | 5.0 | - | 17.0 |  | 9.0 | 5.0 | - | 13.0 |  | 17.0 | 8.0 | - | 18.0 |  | 0.173 | 0.507 |
| NIHSS-improvement | 10.0 | 4.0 | - | 21.0 |  | 9.0 | 4.0 | - | 14.0 |  | 6.0 | 2.0 | - | 17.0 |  | 0.545 | 0.289 |
| mRS-admission | 4.0 | 4.0 | - | 5.0 |  | 4.0 | 4.0 | - | 5.0 |  | 4.0 | 4.0 | - | 5.0 |  | 0.183 | 0.370 |
| mRS-1 year later | 2.0 | 2.0 | - | 5.0 |  | 2.0 | 2.0 | - | 4.0 |  | 2.0 | 1.0 | - | 5.0 |  | 0.404 | 0.181 |
| mRS-improvement | 1.0 | 0.0 | - | 3.0 |  | 2.0 | 0.0 | - | 3.0 |  | 2.0 | 0.0 | - | 4.0 |  | 0.649 | 0.372 |
| Barthel index-admission | 15.0 | 0.0 | - | 20.0 |  | 10.0 | 10.0 | - | 20.0 |  | 10.0 | 5.0 | - | 20.0 |  | 0.410 | 0.576 |
| Barthel index-1 year later | 35.0 | 10.0 | - | 45.0 |  | 30.5 | 5.0 | - | 45.0 |  | 20.0 | 0.0 | - | 20.0 |  | 0.248 | 0.231 |
| Barthel index-improvement | 15.0 | 0.0 | - | 35.0 |  | 10.0 | 0.0 | - | 25.0 |  | 0.0 | -10.0 | - | 15.0 |  | 0.173 | 0.083 |
| TICA: terminal internal carotid artery; M1: M1 segment of the middle carotid artery (MCA); M2: M2 segment of the MCA; IQR: Percentile 25-75; P-value^a^ by Kruskal Wallis Test; P-value^b^ by Jonckheere Terpstra Test. | | | | | | | | | | | | | | | | | |

**Table** **S2** Baseline demographic of patients with differing occlusion sites: TICA, M1 and M2.

|  |  |  |  |  | Occlusion site | | | | | | | |  |  |  |
| --- | --- | --- | --- | --- | --- | --- | --- | --- | --- | --- | --- | --- | --- | --- | --- |
|  |  | Total (n=92) | |  | Terminal ICA (n=29) | |  | M1 (n=42) | |  | M2 (n=21) | |  | P-value^a^ | P-value^b^ |
|  |  | N | % |  | N | % |  | N | % |  | N | % |  |  |  |
| Gender | Female | 39 | 42.4 |  | 14 | 48.3 |  | 21 | 50.0 |  | 4 | 19.0 |  | 0.048 |  |
|  | Male | 53 | 57.6 |  | 15 | 51.7 |  | 21 | 50.0 |  | 17 | 81.0 |  |  |  |
|  |  |  |  |  |  |  |  |  |  |  |  |  |  |  |  |
| Mixed hyperlipidemia | No | 79 | 85.9 |  | 28 | 96.6 |  | 34 | 81.0 |  | 17 | 81.0 |  | 0.118 | 0.093 |
|  | Yes | 13 | 14.1 |  | 1 | 3.4 |  | 8 | 19.0 |  | 4 | 19.0 |  |  |  |
|  |  |  |  |  |  |  |  |  |  |  |  |  |  |  |  |
| Previous stroke | No | 75 | 81.5 |  | 25 | 86.2 |  | 33 | 78.6 |  | 17 | 81.0 |  | 0.715 | 0.590 |
|  | Yes | 17 | 18.5 |  | 4 | 13.8 |  | 9 | 21.4 |  | 4 | 19.0 |  |  |  |
|  |  |  |  |  |  |  |  |  |  |  |  |  |  |  |  |
| CAD | No | 84 | 91.3 |  | 25 | 86.2 |  | 41 | 97.6 |  | 18 | 85.7 |  | 0.111 | 0.878 |
|  | Yes | 8 | 8.7 |  | 4 | 13.8 |  | 1 | 2.4 |  | 3 | 14.3 |  |  |  |
|  |  |  |  |  |  |  |  |  |  |  |  |  |  |  |  |
| Stroke side | Right | 43 | 46.7 |  | 12 | 41.4 |  | 22 | 52.4 |  | 9 | 42.9 |  | 0.607 |  |
|  | Left | 49 | 53.3 |  | 17 | 58.6 |  | 20 | 47.6 |  | 12 | 57.1 |  |  |  |
|  |  |  |  |  |  |  |  |  |  |  |  |  |  |  |  |
| Intra posterior stenosis | No | 50 | 54.3 |  | 16 | 55.2 |  | 25 | 59.5 |  | 9 | 42.9 |  | 0.454 | 0.451 |
|  | Yes | 42 | 45.7 |  | 13 | 44.8 |  | 17 | 40.5 |  | 12 | 57.1 |  |  |  |
|  |  |  |  |  |  |  |  |  |  |  |  |  |  |  |  |
| MCA stenosis-ipsilateral | No | 28 | 30.4 |  | 6 | 20.7 |  | 10 | 23.8 |  | 12 | 57.1 |  | 0.010 | 0.009 |
|  | Yes | 64 | 69.6 |  | 23 | 79.3 |  | 32 | 76.2 |  | 9 | 42.9 |  |  |  |
|  |  |  |  |  |  |  |  |  |  |  |  |  |  |  |  |
| MCA stenosis-contralateral | No | 62 | 67.4 |  | 24 | 82.8 |  | 33 | 78.6 |  | 5 | 23.8 |  | <0.001 | <0.001 |
|  | Yes | 30 | 32.6 |  | 5 | 17.2 |  | 9 | 21.4 |  | 16 | 76.2 |  |  |  |
|  |  |  |  |  |  |  |  |  |  |  |  |  |  |  |  |
| Stroke location | Cortex | 15 | 16.3 |  | 3 | 10.3 |  | 3 | 7.1 |  | 9 | 42.9 |  | 0.001 | 0.001 |
|  | Deep | 19 | 20.7 |  | 4 | 13.8 |  | 9 | 21.4 |  | 6 | 28.6 |  |  |  |
|  | Combined | 58 | 63.0 |  | 22 | 75.9 |  | 30 | 71.4 |  | 6 | 28.6 |  |  |  |
|  |  |  |  |  |  |  |  |  |  |  |  |  |  |  |  |
| Collateral flow-ipsilateral | No | 45 | 48.9 |  | 14 | 48.3 |  | 18 | 42.9 |  | 13 | 61.9 |  | 0.361 | 0.409 |
|  | Yes | 47 | 51.1 |  | 15 | 51.7 |  | 24 | 57.1 |  | 8 | 38.1 |  |  |  |
|  |  |  |  |  |  |  |  |  |  |  |  |  |  |  |  |
| Endovascular mTICI grading | 2a | 30 | 32.6 |  | 7 | 24.1 |  | 17 | 40.5 |  | 6 | 28.6 |  | 0.415 | 0.735 |
|  | 2b | 22 | 23.9 |  | 10 | 34.5 |  | 7 | 16.7 |  | 5 | 23.8 |  |  |  |
|  | 3 | 40 | 43.5 |  | 12 | 41.4 |  | 18 | 42.9 |  | 10 | 47.6 |  |  |  |
|  |  |  |  |  |  |  |  |  |  |  |  |  |  |  |  |
| Cerebral bleeding-follow up | No | 69 | 75.0 |  | 22 | 75.9 |  | 35 | 83.3 |  | 12 | 57.1 |  | 0.077 | 0.191 |
|  | Yes | 23 | 25.0 |  | 7 | 24.1 |  | 7 | 16.7 |  | 9 | 42.9 |  |  |  |
|  |  |  |  |  |  |  |  |  |  |  |  |  |  |  |  |
| Cerebral bleeding grading | HI1 | 13 | 56.5 |  | 5 | 71.4 |  | 3 | 42.9 |  | 5 | 55.6 |  | 0.811 | 0.444 |
|  | HI2 | 6 | 26.1 |  | 2 | 28.6 |  | 2 | 28.6 |  | 2 | 22.2 |  |  |  |
|  | PH1 | 3 | 13.0 |  | 0 | 0.0 |  | 1 | 14.3 |  | 2 | 22.2 |  |  |  |
|  | PH2 | 1 | 4.3 |  | 0 | 0.0 |  | 1 | 14.3 |  | 0 | 0.0 |  |  |  |
|  |  |  |  |  |  |  |  |  |  |  |  |  |  |  |  |
| NIHSS improved | No | 11 | 12.0 |  | 4 | 13.8 |  | 3 | 7.1 |  | 4 | 19.0 |  | 0.312 | 0.676 |
|  | Yes | 81 | 88.0 |  | 25 | 86.2 |  | 39 | 92.9 |  | 17 | 81.0 |  |  |  |
|  |  |  |  |  |  |  |  |  |  |  |  |  |  |  |  |
| mRS improved | No | 28 | 30.4 |  | 11 | 37.9 |  | 11 | 26.2 |  | 6 | 28.6 |  | 0.559 | 0.430 |
|  | Yes | 64 | 69.6 |  | 18 | 62.1 |  | 31 | 73.8 |  | 15 | 71.4 |  |  |  |
|  |  |  |  |  |  |  |  |  |  |  |  |  |  |  |  |
| Barthel index improved | No | 37 | 40.2 |  | 11 | 37.9 |  | 15 | 35.7 |  | 11 | 52.4 |  | 0.425 | 0.353 |
|  | Yes | 55 | 59.8 |  | 18 | 62.1 |  | 27 | 64.3 |  | 10 | 47.6 |  |  |  |
| P-value^a^ by Chi-square test or Fisher's exact test when appropriated; P-value^b^ by Chi-square test for trend; CAD: coronary artery disease; MCA: middle cerebral artery; mTICI: The modified thrombolysis in cerebral infarction; NIHSS: National Institute of Health Stroke Scale; mRS: modified Rankin Scale. | | | | | | | | | | | | | | | |

**Table** **S3** Outcome measurements at baseline and one year post-procedure.

|  |  | NIHSS-1 year later | | | | |  |  |  |  |
| --- | --- | --- | --- | --- | --- | --- | --- | --- | --- | --- |
|  |  | >3 | |  | <=3 | |  | Total | | P-value |
|  |  | N | % |  | N | % |  | N | % |  |
| NIHSS-admission | >3 | 73 | 79.3 |  | 18 | 19.6 |  | 91 | 98.9 | <0.001 |
|  | <=3 | 0 | 0.0 |  | 1 | 1.1 |  | 1 | 1.1 |  |
| Total |  | 73 | 79.3 |  | 19 | 20.7 |  | 92 | 100.0 |  |
|  |  |  |  |  |  |  |  |  |  |  |
|  |  | mRS-1 year later | | | | |  |  |  |  |
|  |  | >2 | |  | <=2 | |  | Total | | P-value |
|  |  | N | % |  | N | % |  | N | % |  |
| mRS-admission | >2 | 47 | 51.1 |  | 44 | 47.8 |  | 91 | 98.9 | <0.001 |
|  | <=2 | 0 | 0.0 |  | 1 | 1.1 |  | 1 | 1.1 |  |
| Total |  | 47 | 51.1 |  | 45 | 48.9 |  | 92 | 100.0 |  |
|  |  |  |  |  |  |  |  |  |  |  |
|  |  | Barthel index-1 year later | | | | |  |  |  |  |
|  |  | <95 | |  | >=95 | |  | Total | | P-value |
|  |  | N | % |  | N | % |  | N | % |  |
| Barthel index-admission | <95 | 84 | 91.3 |  | 8 | 8.7 |  | 92 | 100.0 | <0.001 |
|  | >=95 | 0 | 0.0 |  | 0 | 0.0 |  | 0 | 0.0 |  |
| Total |  | 84 | 91.3 |  | 8 | 8.7 |  | 92 | 100.0 |  |
| P-value by McNemar Test; NIHSS: National Institue of Health Stroke Scale; mRS: modified Rankin Scale. | | | | | | | | | | |
